# Trends in type 2 diabetes incidence and mortality in Scotland between 2004 and 2013

**DOI:** 10.1007/s00125-016-4054-9

**Published:** 2016-07-28

**Authors:** Stephanie H. Read, Joannes J. Kerssens, David A. McAllister, Helen M. Colhoun, Colin M. Fischbacher, Robert S. Lindsay, Rory J. McCrimmon, John A. McKnight, John R. Petrie, Naveed Sattar, Sarah H. Wild, On behalf of the Scottish Diabetes Research Network Epidemiology Group

**Affiliations:** 1 Usher Institute of Population Health Sciences and Informatics, University of Edinburgh, Teviot Place, Edinburgh, EH8 9AG UK; 2 Information Services Division, NHS National Services Scotland, Edinburgh, UK; 3 Institute of Genetics and Molecular Medicine, University of Edinburgh, Edinburgh, UK; 4 Institute of Cardiovascular and Medical Sciences, University of Glasgow, Glasgow, UK; 5 Division of Cardiovascular & Diabetes Medicine, University of Dundee, Dundee, UK; 6 Metabolic Unit, Western General Hospital, Edinburgh, UK

**Keywords:** Epidemiology, Prediction and prevention of type 2 diabetes, Socioeconomic aspects

## Abstract

**Aims/hypothesis:**

The relative contribution of increasing incidence and declining mortality to increasing prevalence of type 2 diabetes in Scotland is unclear. Trends in incidence and mortality rates are described for type 2 diabetes in Scotland between 2004 and 2013 by age, sex and socioeconomic deprivation.

**Methods:**

Data for incident and prevalent cases of type 2 diabetes were obtained from the Scottish national diabetes register with number of deaths identified from linkage to mortality records. Population size and death data for Scotland by age, sex and socioeconomic deprivation were obtained from National Records of Scotland. Age- and sex-specific incidence and mortality rates stratified by year and deciles of socioeconomic status were calculated using Poisson models.

**Results:**

There were 180,290 incident cases of type 2 diabetes in Scotland between 2004 and 2013. Overall, incidence of type 2 diabetes remained stable over time and was 4.88 (95% CI 4.84, 4.90) and 3.33 (3.28, 3.32) per 1000 in men and women, respectively. However, incidence increased among young men, remained stable in young women, and declined in older men and women. Incidence rates declined in all socioeconomic groups but increased after 2008 in the most deprived groups. Standardised mortality ratios associated with diabetes, adjusted for age and socioeconomic group, were 1.38 (1.36, 1.41) in men and 1.49 (1.45, 1.52) in women, and remained constant over time.

**Conclusions/interpretation:**

Incidence of type 2 diabetes has stabilised in recent years suggesting that increasing prevalence may be primarily attributed to declining mortality. Prevention of type 2 diabetes remains important, particularly among socioeconomically deprived populations.

**Electronic supplementary material:**

The online version of this article (doi:10.1007/s00125-016-4054-9) contains peer-reviewed but unedited supplementary material, which is available to authorised users.

## Introduction

Changing distribution of population demographic and anthropometric characteristics, improved diabetes detection and survival have contributed to the growing prevalence of type 2 diabetes in high-income countries. The health and economic implications of these trends are likely to be substantial with recent estimates from the International Diabetes Federation suggesting that European countries spent approximately USD 156 billion on diabetes healthcare in 2015, a figure projected to increase to USD 174 billion by 2040 [1].

The prevalence of all types of diabetes increased from 3.2% to 5.1% in Scotland between 2004 and 2013 [2]. In populations with stable distributions of demographic characteristics, time trends in prevalence of diabetes are influenced by the balance between changes in incidence and mortality. An understanding of recent trends in type 2 diabetes incidence and mortality is necessary to assess health inequalities, to evaluate existing approaches and to plan new approaches for both the prevention and treatment of diabetes.

To achieve the greatest reductions in absolute incidence and mortality, interventions need to target the populations at highest risk. This requires an understanding of whether trends in incidence and mortality are consistent across age, sex and socioeconomic groups. Previous studies have reported higher incidence and mortality among men and the most deprived socioeconomic groups but contemporary data are lacking, particularly in Scotland [3–5]. It is not clear whether the intensification of risk factor management during the last decade among people with type 2 diabetes has reduced disparities in type 2 diabetes mortality across different groups.

The aim of this work was to describe the contribution of changes in incidence and mortality rates to increasing prevalence of type 2 diabetes in Scotland between 2004 and 2013, and to investigate whether trends were similar across age, sex and socioeconomic groups.

## Methods

### Data sources

Population-based data for people with diagnosed diabetes in Scotland were obtained from a 2014 extract of the Scottish Care Information-Diabetes (SCI-Diabetes) database. Briefly, this database was established at a national level in 2000, and contains demographic and clinical data relevant to diabetes care. It is populated by daily downloads from primary and secondary care databases across Scotland with increasing completeness since 2004. As of 2012, data from all of the 997 general practices in Scotland have been included in the SCI-Diabetes database. A validation study among the subset of people with diabetes mentioned on a hospital record in 2007 found that 99% were included in the diabetes register [6]. Within the SCI-Diabetes database, diabetes type is available as a clinician-recorded variable. Following a diagnosis of diabetes (type 1, type 2, MODY, gestational, etc.), clinicians record diabetes type using a drop-down menu within electronic health records. In primary care, these are coded using the Read code system. To improve the accuracy of this variable for research purposes, an algorithm which combines information from the clinician-recorded diabetes type variable and prescription data is used. More specifically, patients are assigned to a diabetes type based on their use of sulfonylureas for longer than a year and/or the timing of insulin use in relation to diagnosis date, in combination with the clinician-recorded variable. We chose to use sulfonylureas because of the increasing use of other classes of non-insulin glucose lowering drugs in people with type 1 diabetes. The use of this algorithm results in a 1.5% increase in the number of people defined as having type 2 diabetes from the original clinician-recorded variable, primarily through the reclassification of people who had a recording of ‘unknown diabetes’ or ‘type 1 diabetes’ [7]. Incidence of type 2 diabetes was estimated by age, sex, Scottish Index of Multiple Deprivation (SIMD) decile and calendar year using population denominators based on numbers of people without diabetes, identified by subtraction of the numbers of people with diabetes from total population estimates. Mid-year population estimates and numbers of deaths each year (2004–2013), age in one-year classes, sex and deciles of SIMD were obtained from National Records Scotland (NRS). The mid-year population was used as an estimate of the person-years in the population for each year. The SIMD is an area-based measure of socioeconomic status that uses information from seven domains including income, education and crime to assign deprivation scores to 6505 data-zones across Scotland [8]. These data-zones are ranked according to their deprivation score and divided using deciles, with decile 1 representing the 10% most deprived areas and decile 10 representing the 10% least deprived areas.

Follow-up time for people with type 2 diabetes was calculated from date of diagnosis of diabetes until date of death or 31st December 2013, whichever came first. Follow-up time was truncated at age 90 years since NRS population estimates were not available in yearly age groups for persons over 90 years. As type 2 diabetes is rare in younger people the study was based on people over 39 years of age. Numbers of deaths and time at risk were tabulated by calendar year, age, sex, year of birth, deprivation decile and diabetes duration.

To calculate risk time in person-years for the non-diabetic comparison group, risk time among persons with diabetes was subtracted from mid-year population estimates for Scotland by age, sex and deprivation decile. Number of deaths in people without type 2 diabetes were calculated using a similar method of subtraction of deaths among people with diabetes from national data.

Approval for the creation and analysis of the linked dataset containing no personal identifying information was obtained from the SCI-Diabetes Collaboration steering committee, the Scottish multicentre research ethics committee (reference number 11/AL/0225), the Privacy Advisory Committee of NHS National Services Scotland (reference 33/11) and Caldicott Guardians of all health boards.

### Statistical methods

All analyses were conducted separately for men and women. Directly age-standardised incidence and mortality rates by year of diagnosis were calculated using the 2013 European Standard Population in order to adjust for confounding by age and to facilitate comparison with other populations.

Poisson models were used to estimate standardised incidence and mortality rates by age, calendar year and deprivation decile as smooth functions using natural cubic splines. Standardised mortality ratios (SMRs) to compare mortality rates in people with type 2 diabetes with the non-diabetic population were modelled using Poisson models including an indicator for diabetes status and adjustment for age, calendar year and deprivation as smooth functions using natural cubic splines. The validity of this approach was checked by investigating the deviance of models using deprivation coded in different ways and the use of splines was found to be appropriate. Data for single year age groups within decades and selected deprivation deciles are used for illustration. Rates are expressed as events per 1000 person years. Interaction terms between age, time and deprivation were included in models where these improved the goodness of fit. All analyses were conducted in R [9] (www.R-project.org) and all tabulations were generated using the Epi package [10].

## Results

At the time of data extraction, data for 398,076 people with a diagnosis of diabetes, regardless of vital status, were available on the SCI-Diabetes database. Of these, 7405 (1.9%) had incomplete data on deprivation status and/or date of diagnosis and were excluded. The mid-year estimate of the population of Scotland for 2008 was 5,168,500.

Between 2004 and 2013, 180,290 people aged between 40 and 89 years were diagnosed with type 2 diabetes in Scotland, and the proportion of men among incident cases increased from 53% to 57% (Table 1). Age-standardised incidence rates changed little over time, particularly from 2005 onwards.

**Table 1:** Number of incident cases of type 2 diabetes and incidence rates directly standardised to the 2013 European Standard Population in the Scottish Diabetes Registry by year of diagnosis (2004–2013)

	Men with type 2 diabetes		Women with type 2 diabetes		Total with type 2 diabetes	
Year	Cases	Incidence	Cases	Incidence	Cases (% men)	Incidence
2004	9931	5.10	8889	3.80	18,820 (52.8)	4.39
2005	9513	4.82	8096	3.45	17,609 (54.0)	4.08
2006	9595	4.80	7833	3.31	17,428 (55.1)	4.00
2007	9753	4.78	7685	3.22	17,438 (55.9)	3.95
2008	10,139	4.94	7894	3.29	18,033 (56.2)	4.05
2009	10,445	5.03	8135	3.36	18,580 (56.2)	4.13
2010	10,373	4.94	7893	3.23	18,266 (56.8)	4.02
2011	9880	4.64	7651	3.10	17,531 (56.4)	3.82
2012	10,457	4.85	7783	3.14	18,240 (57.3)	3.94
2013	10,493	4.88	7852	3.14	18,345 (57.2)	3.94
Total	100,579	4.88	79,711	3.30	180,290 (55.8)	4.03

Cases, number of cases; incidence, incidence rate per 1000 (age-standardised to the 2013 European Standard Population)

**Fig. 1:**
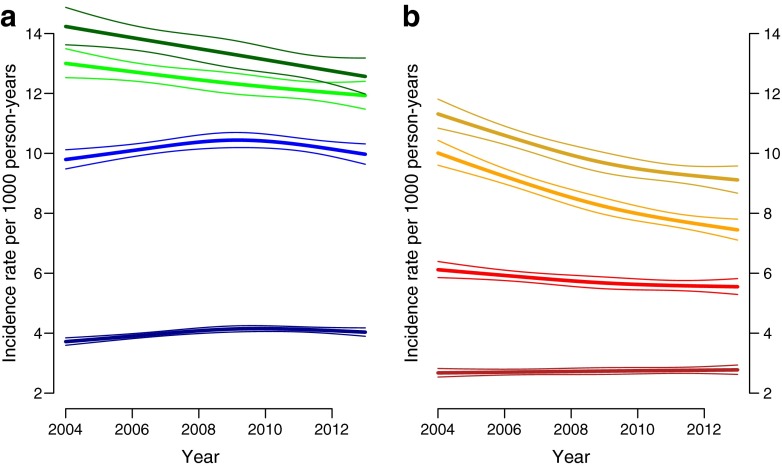
Age-specific trends in incidence rates of type 2 diabetes among people in deprivation decile 5 in Scotland between 2004 and 2013 for (**a**) men (ages: dark green, 75 years; light green, 65 years; light blue, 55 years; dark blue, 45 years) and (**b**) women (ages: dark yellow, 75 years; light yellow, 65 years; light red, 55 years; dark red, 45 years)

For both men and women, incidence rates were highest at 75 years of age and lowest at 45 years of age with similar patterns observed in all of the deprivation deciles (data for deprivation decile 5 shown in Fig. 1). Incidence rates increased slightly over the study period in 45-year-old women but declined in older women. Between 2004 and 2009, incidence rates increased in men aged 45 and 55 years but declined after 2009. In older men, incidence rates declined during the study period.

Overall, there were 10,508 diagnoses of type 2 diabetes during 25.3 million person-years of follow-up among people aged below 40 years. Due to small event numbers in individual years, stratification of incidence rates by sex or deprivation was not possible. However, there was no clear gradient in crude incidence rates between 2004 and 2013 with incidence rates varying between 0.38 diagnoses per 1000 person-years to 0.46 diagnoses (ESM Table 1).

**Fig. 2:**
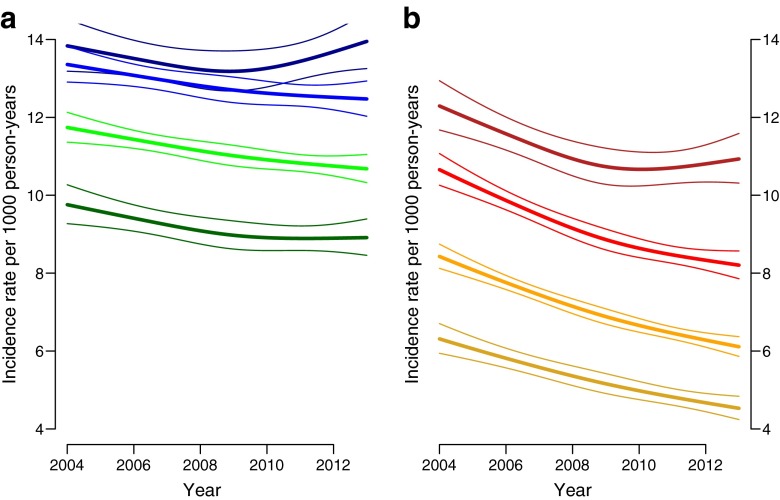
Trends in incidence rates by deprivation deciles among people aged 65 years for (**a**) men (deprivation deciles: dark blue, D1 [most deprived]; light blue, D4; light green, D7; dark green, D10 [least deprived]) and (**b**) women (deprivation deciles: dark red, D1 [most deprived]; light red, D4; light yellow, D7; dark yellow, D10 [least deprived])

Incidence rates were higher among more deprived groups with a more marked effect of deprivation among women than men (Fig. 2). Incidence declined across all deprivation categories for men and women but the decline was slower among more deprived deciles. Furthermore, from around 2010, incidence of type 2 diabetes appeared to increase among men and women in the most deprived decile, leading to widening inequality in diabetes incidence in later years.

There were 77,953 deaths during 1,910,894 person-years of follow-up among people with type 2 diabetes between 2004 and 2013 (Table 2). There were more incident cases of type 2 diabetes (n = 180,290) than deaths leading to an increase of 102,337 people with type 2 diabetes during the study period.

**Table 2:** Number of deaths, age-standardised mortality rates and number of surplus type 2 diabetes cases among men and women with type 2 diabetes in Scotland by year

	Men with type 2 diabetes		Women with type 2 diabetes		Total with type 2 diabetes		New cases - deaths
Year	Deaths	Mortality	Deaths	Mortality	Deaths (% men)	Mortality	
2004	3040	20.8	2839	18.8	5879 (51.7)	19.8	12,941
2005	3407	21.7	3182	17.1	6589 (51.7)	19.2	11,020
2006	3545	21.4	3277	16.6	6822 (52.0)	18.7	10,606
2007	3875	20.4	3505	16.7	7380 (52.5)	18.4	10,058
2008	4020	19.3	3741	17.0	7761 (51.8)	18.2	10,272
2009	4104	19.1	3692	15.5	7796 (52.6)	17.2	10,784
2010	4491	19.1	3893	15.7	8384 (53.6)	17.3	9882
2011	4582	19.3	4189	15.7	8771 (52.2)	17.3	8760
2012	4812	18.8	4422	17.4	9234 (52.1)	18.3	9006
2013	4993	18.4	4344	15.2	9337 (53.5)	16.7	9008
Total	40,869	19.8	37,084	16.6	77,953 (52.4)	18.1	102,337

Deaths, number of deaths; mortality, mortality rate per 1000 (age-standardised to the 2013 European Standard Population)

Overall, age-standardised mortality rates declined by 11.5% for men and 15.7% for women between 2004 and 2013 with similar declines across age groups and deprivation deciles (ESM Figs 1, 2).

The overall excess risk of death among men and women with type 2 diabetes was approximately 40% and 80%, respectively, compared with people without diabetes. SMRs increased slightly between 2004 and 2013 but this change was not statistically significant (ESM Fig. 3). Younger people had the highest SMRs and the largest differences in risk by age were observed among women (ESM Fig. 4). SMRs were highest in the least deprived groups but changes in SMR risks over time were similar across deprivation deciles (ESM Fig. 5). There were larger differences in SMRs between the most and least deprived men than for women, for whom SMRs were higher than for men in all SIMD deciles.

## Discussion

Using this population-based register of people with diabetes in Scotland, we have reported contemporary rates and trends in type 2 diabetes incidence and mortality between 2004 and 2013 and examined the effect of age, sex and socioeconomic deprivation on these estimates.

Type 2 diabetes incidence was found to be relatively stable after 2004 while mortality rates declined. The number of new cases of type 2 diabetes exceeded the number of diabetes deaths. Important differences in trends in incidence and mortality rates were observed by age, sex and socioeconomic status. Despite improvements in absolute mortality rates, type 2 diabetes continues to confer an excess risk of death compared with the non-diabetic population.

### Relation to other studies

Declining or stable type 2 diabetes incidence in the mid-2000s has also been observed in other populations [5, 11–13]. A register-based study in Denmark, which was unable to distinguish by diabetes type, reported increasing diabetes incidence between 1995 and 2004 but declining incidence between 2004 and 2007 [11]. In Sweden, the incidence of pharmacologically treated diabetes decreased modestly between 2005 and 2013 [13]. The National Health Interview Survey in the USA identified that diabetes incidence doubled between 1990 and 2008 before plateauing between 2008 and 2012 [12]. Type 2 diabetes incidence stabilised after 2005 in the UK according to data from The Health Improvement Network (THIN) database [5].

In contrast, another UK-based study conducted using the Clinical Practice Research Datalink database reported that standardised incidence ratios of diabetes increased steadily from 100 (referent period) between 1991 and 1995 to 275 (273–276) between 2006 and 2010 [14]. Increasing age-standardised type 2 diabetes incidence among men participating in the British Regional Heart Study was observed between 1986 and 2007 [15]. These conflicting findings may reflect differences in diabetes definition, dates of comparison, methods of calculating disease incidence and underlying population characteristics.

The largest fall in type 2 diabetes incidence in Scotland took place between 2004 and 2005. The introduction of the Quality and Outcomes Framework (QOF) in the UK in 2004, which provided incentives for general practitioners to have a diabetes register, may have contributed to this finding.

A proposed explanation for declining or stable diabetes incidence after 2005 is a reduction in the pool of undiagnosed diabetes through the intensification of diagnostic activities during the last decade [11, 16]. In 1998, the World Health Organization altered the diagnostic criteria by lowering the threshold for diagnosing diabetes [17], a factor which may have contributed to the marked increases in diabetes incidence in the late 1990s and early 2000s. For example, type 2 diabetes incidence doubled between 1993 and 2004 in Tayside, Scotland. This increase may partly reflect improved diagnosis of diabetes in addition to the change in diagnostic criteria [18]. This proposed explanation is also supported by several studies which have reported declining numbers of undiagnosed diabetes cases during the last 20 years [19–21]. Data from the German Health Interview and Examination Surveys indicate that the proportion of undiagnosed diabetes decreased from 40% in 1997 to 22% in 2010 [19]. Furthermore, the proportion of diabetes cases which were undiagnosed in Scotland declined from 17% in 2010 to 12% in 2013 according to estimates from the Association of Public Health Observatories diabetes prevalence model, although it is not known if the proportion was higher in the early 2000s [22]. Our finding of differences in incidence trends by age may reflect different patterns of opportunistic screening and obesity trends.

Stable prevalence of adulthood obesity, an established risk factor for type 2 diabetes, is also likely to have influenced our finding of stable diabetes incidence [23]. Estimates from the Scottish Health Surveys indicate that the proportion of adults who were obese increased from 24.2% in 2003 to 27.1% in 2009 but remained constant thereafter. Moreover, trends in age-specific obesity prevalence mirror trends in age-specific diabetes incidence [23]. For example, data from the Scottish Health Surveys suggest that the proportion of obese men aged 35–44 years peaked in 2009 at 31.9% before declining to 24.7% by 2013; a similar pattern was observed in men aged 45–54 years. This trend coincides with a peak in diabetes incidence in 2009 before declining thereafter among men aged below 65 years.

The disparity in incidence rates between men and women has been observed in previous studies [11, 16]. One explanation for this is the higher risk associated with male body fat distribution and greater insulin resistance among men relative to women [24]. Findings from a study conducted using the SCI-Diabetes dataset support this notion with men developing type 2 diabetes at lower BMIs than women of a similar age, with particularly marked differences at younger ages [25]. Deprivation was also strongly associated with incidence of type 2 diabetes and socioeconomic inequalities in incidence of type 2 diabetes widened during the study period, particularly among women. Data from the Whitehall Study II suggest that health behaviours and BMI explained up to 45% of the differences in type 2 diabetes incidence by socioeconomic status [26]. These findings underline the importance of targeting efforts to improve levels of modifiable risk factors in more deprived groups to achieve reductions in type 2 diabetes incidence and health inequalities.

Our finding of declining absolute mortality among people with type 2 diabetes in recent years corresponds with results from several studies conducted in developed countries, including Canada [16], the UK [27], Denmark [11, 28], Australia [29] and the USA [30]. Increased testing may have led to earlier diagnosis of diabetes or diagnosis of people with different characteristics from those diagnosed in the early 2000s. However, while previous studies have reported declining values of glycated haemoglobin at diagnosis during this study period [31], there was no evidence of a downward trend in glycated haemoglobin levels at diagnosis of type 2 diabetes by year using data from SCI-Diabetes (see ESM Table 2).

Improved management of hypertension, cholesterol and diabetes, as well as reductions in smoking, are also likely to have contributed to the observed declines in mortality rates. During the last decade, diabetes treatment guidelines have emphasised the need for intensive risk factor control [32, 33], while the implementation of the QOF has contributed to improved diabetes care and monitoring. Indeed, the 2014 Scottish Diabetes Survey reported that the proportion of people with type 2 diabetes and a record of HbA_1c_ below 7.5% (58 mmol/mol), reflecting good diabetes control, increased from 50% to 61% between 2004 and 2013 [2]. Similarly, the proportion of people with diabetes (not stratified by type 1 and 2) with a systolic blood pressure below 140 mmHg increased from 63% to 79% between 2004 and 2013 [2].

However, if the improvements in mortality represent improved diabetes care or earlier diagnoses of type 2 diabetes then it might be expected that the SMRs in people with type 2 diabetes compared with people without type 2 diabetes would also have declined. We found no change in SMRs during the study period, with mortality rates remaining approximately 40% and 80% higher among men and women with type 2 diabetes, respectively, relative to people without diabetes. These findings contrast with earlier studies which have reported declining SMRs over time and a smaller overall relative influence of type 2 diabetes on mortality [11, 27, 29, 30]. For example, in Australia, SMRs declined from 1.40 (95% CI: 1.36, 1.44) in 1997 to 1.21 (1.19, 1.23) in 2010 in men, and from 1.56 (1.51, 1.61) to 1.22 (1.19, 1.24) in women [29]. Findings from a study using the THIN database reported declining age and sex adjusted SMRs between 1996 and 2009, falling from 2.14 (1.97, 2.32) to 1.65 (1.57, 1.72) [27]. In a Swedish registry study, excess risk of mortality was 27% between 1998 and 2011 after adjusting for age and sex, decreasing to only 15% when additionally adjusting for country of birth, educational level and comorbidity [34]. A time interaction was also reported in this study with type 2 diabetes conferring a 17% excess risk of mortality compared with controls matched for age, sex and county between 1998 and 2001, a figure which declined to 13% between 2005 and 2011. Explanations for lower excess risks of mortality among people with type 2 diabetes in these countries compared with Scotland include methodological differences between the studies such as additional adjustment for confounders, smaller differences in the health status between people with and without diabetes or differences in diabetes care.

Our findings of marked sex differences in SMRs associated with type 2 diabetes (See ESM Table 3) is in contrast to findings from earlier studies [11, 27, 29, 30]. Unfortunately, only one of these studies reports absolute mortality rates by sex and diabetes status, thus making it difficult to determine whether the differences in SMRs are primarily driven by absolute mortality among women with type 2 diabetes or mortality among the non-diabetic population. Further investigation is required to identify explanations for higher SMRs associated with type 2 diabetes both in comparison to other settings and between women and men in Scotland.

Mortality rates were higher in the most deprived groups compared the least deprived groups. Similar patterns have been observed in several earlier studies [3, 35–38]. We have shown that socioeconomic inequalities in mortality have persisted throughout the study period. The explanation for poorer survival among the most deprived groups is likely to include differences in risk factor prevalence and control, and healthcare provision and use. In the Scottish diabetes register, the mean age of people with newly diagnosed type 2 diabetes was 59.2 years among the most deprived decile compared with 63.1 years in the least deprived decile. This variance is probably related to differences in obesity prevalence by deprivation (62.4% and 51.5% of obese people in deprivation deciles 1 and 10, respectively). A previous study among people with type 2 diabetes in Scotland found the expected differences in smoking habits but no differences in cholesterol control, blood pressure or glycaemic control by socioeconomic status [39].

### Strengths and weaknesses

Our study has a number of strengths. First, it utilises a large population register that includes over 99% of people with a diagnosis of diabetes in Scotland, allowing presentation of national level estimates that are unlikely to have been heavily influenced by selection bias. Data linkage to death registrations enabled examination of trends in mortality. The use of a robust measure of deprivation also enabled the comprehensive description of the influence of deprivation on trends in incidence and mortality among people with type 2 diabetes which has not been described in previous studies. There is potential for misclassification bias associated with the use of an area-based measure of socioeconomic status but the small size of the data-zones (approximately 800 people) to which deprivation status is assigned means that this is likely to have a small effect.

Our study has some limitations. Our reliance on data collected routinely means that the accuracy of the data cannot be fully verified. In particular, it is possible that some people with type 1 diabetes were misclassified as having type 2 diabetes and subsequently included in the analyses presented here. However, the number of misclassified patients is likely to be very small and a diabetes type algorithm was used to validate type of diabetes, rather than relying on clinical diagnosis alone [7]. We were unable to examine the influence of ethnicity on incidence and mortality rates since accurate estimates of the population distribution by ethnicity are only available for census years (i.e. 2011 for this study period). However, over 90% of people with and without diabetes in Scotland are of white ethnicity and, therefore, our estimates reflect diabetes incidence and mortality for the majority of the population [2]. Furthermore, as data were not available for several risk factors, including the presence of comorbid conditions, in the non-diabetic population, we were unable to investigate possible explanations for the excess risk of mortality among people with type 2 diabetes. The role of risk factor control on mortality among people with diabetes will be investigated in subsequent studies. Finally, data on date of immigration or emigration were not available for these analyses and this limitation may have had a small influence on our estimates.

### Implications

Our findings support the notion that stabilising obesity prevalence and the potentially smaller numbers of people with undiagnosed diabetes through intensified diagnostic activities in earlier years have resulted in stable or declining type 2 diabetes incidence in Scotland. Our findings suggest that improved survival is the leading contributor to increasing diabetes prevalence and these trends are likely to have important implications for health services, partly through the possible increased incidence of complications resulting from longer diabetes durations [40]. Further research is required to identify the relative contributions of better treatment of people with type 2 diabetes and differences in characteristics of newly diagnosed patients to improvements in survival.

Despite improvements in absolute mortality rates, type 2 diabetes confers an excess risk of death compared with the non-diabetic population, and this excess risk is higher in Scotland than in other countries. There is still scope to address the increased mortality associated with type 2 diabetes.

Major inequalities by age, sex and socioeconomic status in type 2 diabetes incidence and mortality highlight the need to implement effective approaches to the prevention and treatment of type 2 diabetes that also attempt to address existing inequalities.

## Electronic supplementary material

Below is the link to the electronic supplementary material.

ESM (PDF 234 kb)

## Abbreviations

NRS: National Records Scotland
SCI-Diabetes: Scottish Care Information - Diabetes
SIMD: Scottish Index of Multiple Deprivation
THIN: The Health Improvement Network
