# Supplementary material for: Trends in type 2 diabetes incidence and mortality in Scotland between 2004 and 2013
Source: Diabetologia. 2016 Jul 28;59(10):2106–13. doi: 10.1007/s00125-016-4054-9 (PMC5016553; doi:10.1007/s00125-016-4054-9)
Supplement: Supplementary file 1 — (PDF 234 kb) [file 125_2016_4054_MOESM1_ESM.pdf]

Electronic Supplementary Material – Trends in type 2 diabetes incidence and mortality in Scotland between 2004 and 2013.

TABLES:

ESM Table 1. Trends in crude incidence of type 2 diabetes among people aged below 40 years between 2004 and 2013

Year	Crude incidence rate (per 1,000 person-years)
2004	0.41
2005	0.37
2006	0.38
2007	0.40
2008	0.44
2009	0.42
2010	0.41
2011	0.41
2012	0.46
2013	0.44
Overall	0.42

ESM Table 2. Mean glycated haemoglobin levels within one month of date of diagnosis of type 2 diabetes by year

Year	Mean Glycated Haemoglobin within 1 month of type 2 diabetes diagnosis, mmol/mol (SD)
2004	66.8 (24.1)
2005	68.3 (24.5)
2006	68.9 (25.1)
2007	68.2 (25.2)
2008	67.0 (24.5)
2009	66.3 (24.6)
2010	66.4 (24.6)
2011	67.2 (23.9)
2012	67.2 (23.9)
2013	65.9 (23.0)

FIGURES:

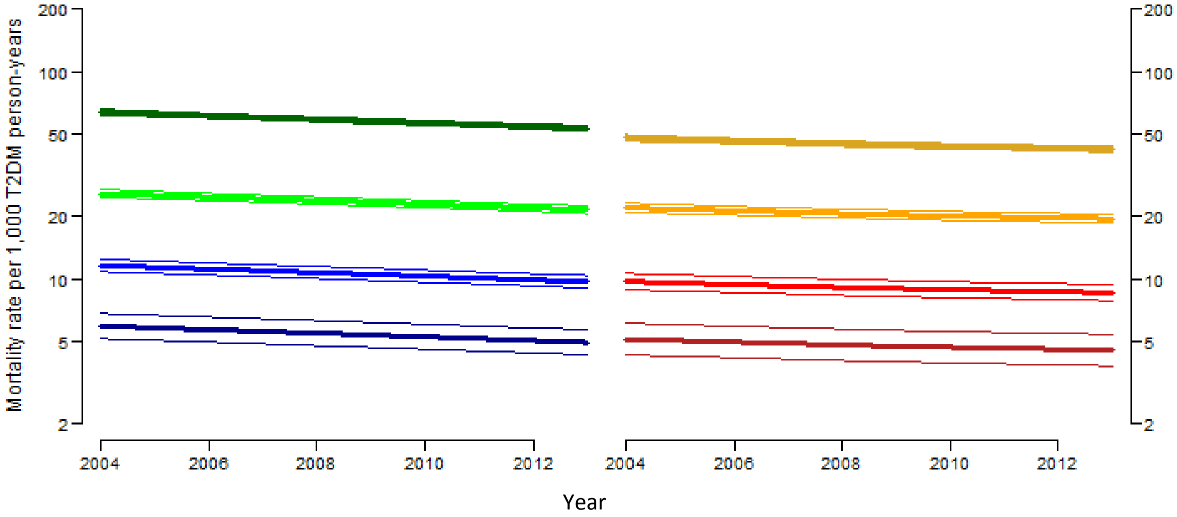

ESM Fig 1. Trends in mortality rates for men (left panel, ages: dark green=75 years, light green=65 years, light blue=55 years, navy blue=45 years) and women (right panel, ages: dark yellow=75 years, light yellow=65 years, light red=55 years, dark red=45 years) in deprivation decile 5 by age.

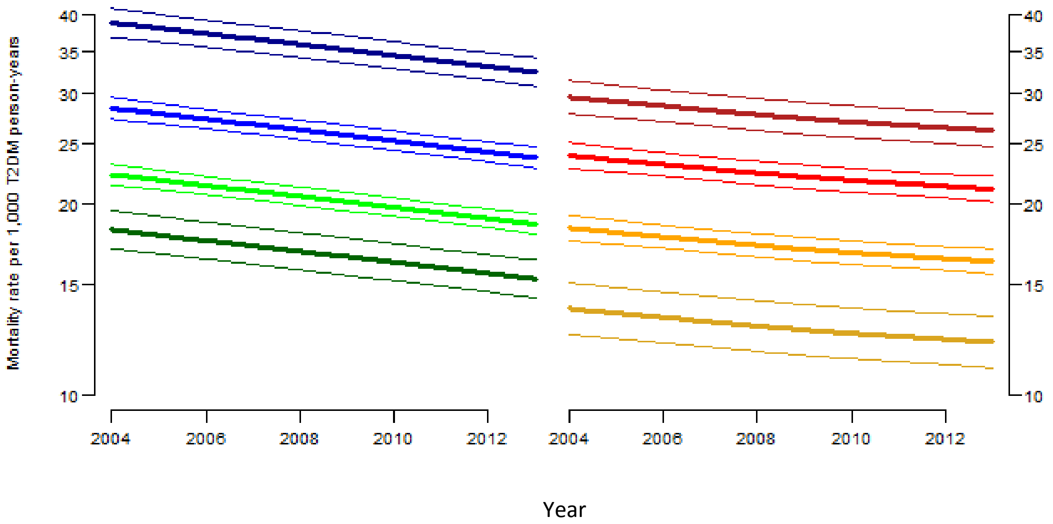

ESM Fig 2. Trends in mortality rates for men (left panel, deprivation deciles: dark blue =D1 (Most deprived), light blue = D4, light green = D7, dark green = Q10(Least deprived) and women (right panel, deprivation deciles: dark red =D1 (Most deprived), light red = D4, light yellow = D7, dark yellow = D10(Least deprived))) aged 65 years by deprivation deciles.

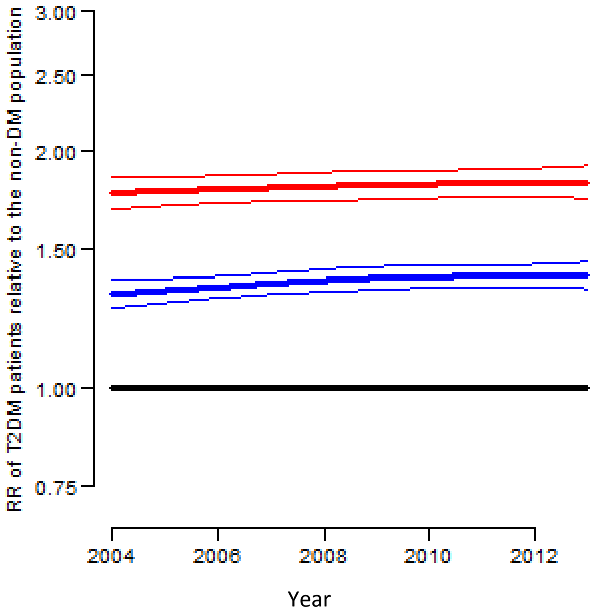

ESM Fig 3. Trends in relative mortality among people with type 2 diabetes compared to people without diabetes between 2004 and 2013 for men (blue line) and women (red line) aged 65 years in deprivation decile 5

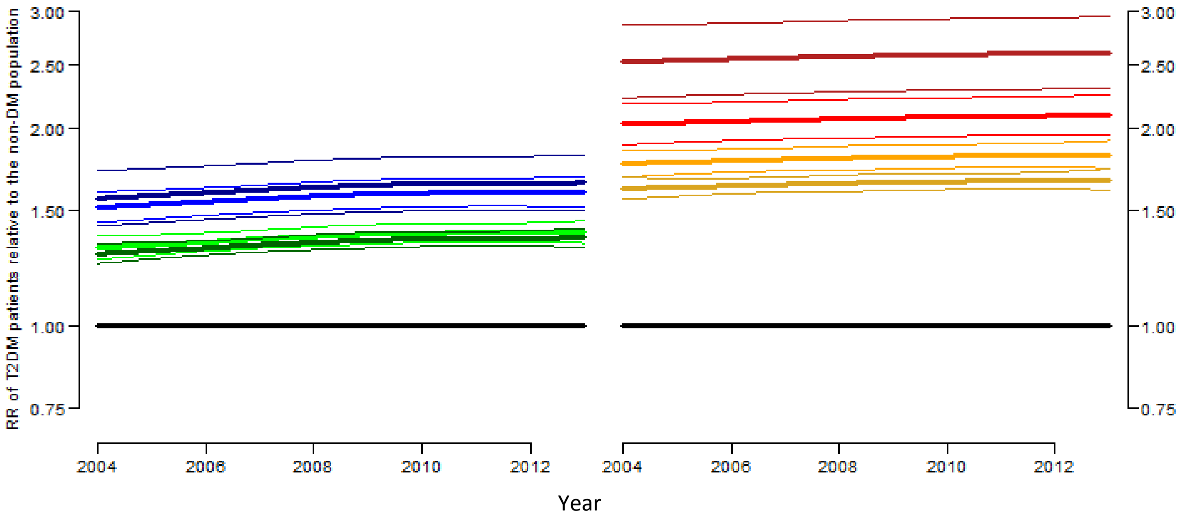

ESM Fig 4. Relative mortality among men (left panel, ages: dark green=75 years, light green=65 years, light blue=55 years, navy blue=45 years) and women (right panel, ages: dark yellow=75 years, light yellow=65 years, light red=55 years, dark red=45 years) with type 2 diabetes in deprivation decile 5 compared to people without diabetes between 2004 and 2013 by age.

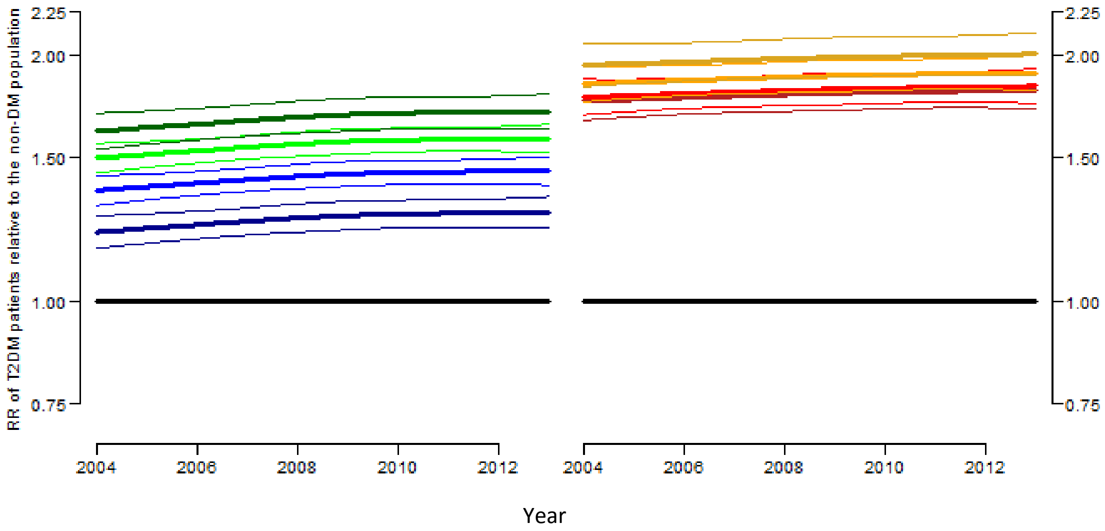

ESM Fig 5. Relative risk of mortality among men (left panel, deprivation deciles: dark blue =D1 (Most deprived), light blue = D4, light green = D7, dark green = D10(Least deprived)) and women (right panel, deprivation deciles: dark red =D1 (Most deprived), light red = D4, light yellow = D7, dark yellow = D10(Least deprived)) with type 2 diabetes compared to people without diabetes between 2004 and 2013 by deprivation and sex.
